# Increasing course speeds in canine agility: a decade of trends from American Kennel Club competition data

**DOI:** 10.3389/fvets.2026.1785106

**Published:** 2026-04-10

**Authors:** Meredith McCormack-Mager, Arielle Pechette Markley, Sarah Fernandezlopez, Abigail B. Shoben

**Affiliations:** 1 Division of Biostatistics, College of Public Health, The Ohio State University, Columbus, OH, United States; 2 Red Sage Integrative Veterinary Partners, Fort Collins, CO, United States; 3 Bad Dog Agility, Pearland, TX, United States

**Keywords:** agility, canine agility, dog agility, dog injury risk, dog sports, performance, speed, veterinary sports medicine

## Abstract

Canine agility is a sport in which handlers direct dogs through pre-set obstacle courses. Over the past two decades, reported injuries among agility dogs have increased, with multiple factors proposed as potential contributors. Anecdotal evidence suggests that course speeds have also risen in recent years, raising concern that faster performance may be associated with greater injury risk. The objective of this study was to determine whether agility competition speeds have increased over the past decade and whether demographic shifts may explain observed changes. Publicly available qualifying run data from the American Kennel Club Masters Jumpers with Weaves class from 2012 to 2024 were analyzed. Trends in qualifying run speed, both overall and within annual cohorts defined by first observed qualifying run at this level, were examined using generalized estimating equations to account for correlation among runs from the same dog. Average qualifying run speed increased by an estimated 0.033 yards per second each year (95% CI: 0.031, 0.036) from 2012 to 2024, with similar trends observed for qualifying runs at the 5th and 95th percentiles. Descriptive cohort analyses demonstrated that average speed increased for several consecutive years within a typical competitive career before reaching a peak and subsequently declining. The average starting speed and average peak speed were higher for more recent cohorts, supporting the hypothesis that agility speeds are increasing. These findings empirically confirm increase in agility competition speeds over time and underscore the importance of future research investigating potential associations between speed and injury risk in agility dogs.

## 1 Introduction

Agility is a sport in which dogs navigate a sequence of obstacles, including jumps, weave poles, tunnels, and contact obstacles (A-frame, dogwalk, teeter), under the direction of a handler. Dogs must complete the obstacle course, which is set by a judge and new to the dog and handler team at each event, within a pre-specified amount of time. Errors, termed “faults,” result in time penalties or disqualification. Competitions, referred to as “trials,” are administered by numerous organizations, each with its own rules and regulations.

The largest such organization in the United States is the American Kennel Club (AKC), whose trials are open to all healthy dogs aged 15 months or older and which reports over 1 million entries to their agility program each year.^1^ At AKC competitions, handlers may choose from a variety of competition types and skill divisions in which to enter their dogs. Dogs are assigned to a jump height category based on their measured height at the withers. Handlers may also choose to compete under “preferred” status, a designation that allows dogs to jump one height category lower and compete with extended time allowances.^2^ While this flexibility provides an opportunity for a broad range of dogs to compete on courses that best suit their needs, handlers have consistently expressed concerns about the safety of participating dogs (1, 2).

Reports of injuries among agility dogs have increased in number over the past two decades (2–4). This increase has occurred despite attempts during this period to improve safety, including greater attention to competition surface characteristics and modification or removal of obstacles perceived as higher risk (3, 5). ^3^^,^ ^4^ Some researchers have hypothesized that increasing competition speeds may contribute to rising injury rates, since faster speeds may increase musculoskeletal load during agility performance (6, 7). While anecdotal reports suggest that agility competition speeds are increasing, no empirical studies have quantified longitudinal changes in speed. To the authors’ knowledge, no published study has evaluated temporal trends in agility competition speed using large-scale competition data.

The objective of this study was to determine whether qualifying run speed increased from 2012 to 2024 using AKC agility trial data. Specifically, temporal trends were evaluated in typical, fastest, and slowest qualifying run speeds, and competition demographics were examined to assess whether shifts in participation could account for observed changes.

## 2 Methods

The data used in this study were obtained from the AKC database on agility trials and are publicly available.^5^ The AKC registers and publishes information on all qualifying runs – runs completed within the pre-specified maximum time for a course and having no more than a specified number of faults – completed at AKC-sponsored agility trials. The AKC does not publish data on non-qualifying runs.

At every AKC agility trial, the trial secretary, under supervision of the trial judge, submits both course-level information (division, course length, and course time) and qualifying dog information (time, jump height, breed, unique AKC number). Course length is estimated by judges, who use a measuring wheel to approximate intended running path, following AKC guidelines, which were unchanged during the study period. All publicly available data from AKC agility trials conducted between 2012 and 2024 were extracted for this study.

Inclusion and exclusion criteria were instituted to ensure relevancy and interpretability of study findings. Analyses were restricted to Masters-level jumpers with weaves (JWW) qualifying runs. Masters is the highest competition level in AKC agility and is therefore the division in which dogs spend the majority of their agility careers. Additionally, runs on Masters courses only qualify if no faults are recorded, thereby reducing the possibility of dogs having longer times due to faults that were then corrected and providing a cleaner estimate of speed over the entire course. JWW courses only include jumps, tunnels, and weave obstacles, thereby reducing some of the variability due to both enforced (i.e., table) and voluntary (i.e., stopped contact criteria) pauses on contact obstacles that occur on “standard” courses.

Regular jump height categories are assigned based on measured height at the withers (Table 1). These jump height categories were unchanged during the entire study period (2012–2024). The “preferred” designation allows any dog, at their handler’s discretion, to jump one height category lower than their regular height category (e.g., a dog that is 12.5″ at the withers would jump 12″ regular or 8″ preferred). AKC generally allows dogs to compete in a jump height higher than their regular height at handler discretion and with no notation on the record, with the exception that dogs shorter than 22″ cannot compete in the regular 24″ class. During the study period, AKC offered a variety of optional higher jumping classes (a 26″ regular class, a 24C class, etc.), and these classes have been excluded from these analyses. Further details on competition formats can be found in AKC’s Regulations for Agility Trials (see text footnote 2).

**Table 1:** Minimum jump height category, by dog height at the withers and preferred status.

Dog height category	Jump height (regular)	Jump height (preferred)
11 inches and under	8 inches	4 inches
11–14 inches	12 inches	8 inches
14–18 inches	16 inches	12 inches
18–22 inches	20 inches	16 inches
Over 22 inches	24 inches	20 inches

Qualifying runs were excluded if any of the following criteria were met: (1) missing information on course distance or result time, (2) implausible course length (entered as 250 yards or greater), (3) presumed inaccurate result times (result time < 10 s or result time greater or equal to one full second over the standard course time).^6^

Generalized estimating equations (GEE) were used to assess changes in average speed over time for all runs, allowing for correlation of runs from the same dog (identified by unique AKC number) (8). All models controlled for preferred status (yes/no) and jump height category and used independence as the working correlation structure. All confidence intervals presented are based on robust standard errors produced by these models. Sensitivity analyses using GEE evaluated temporal trends in the 1% of runs surrounding the 95th, 50th, and 5th percentiles of speed in each calendar year.

Cohort analyses grouped dogs according to the first calendar year in which they achieved a qualifying run in Masters JWW to evaluate longitudinal performance trajectories and potential demographic shifts among new entrants to the division. In subgroup analyses, dogs were assigned to regular or preferred cohorts based on their first qualifying run within the relevant designation.

Descriptive analyses were conducted to evaluate temporal trends in competition characteristics and demographics that might contextualize observed changes in speed. Annual summary statistics were calculated and graphically displayed for jump height distribution, preferred status participation, number of qualifying runs, number of unique competitors, and number of new cohort entrants. Because the dataset represents a census of all publicly available qualifying runs during the study period rather than a sampled subset, formal hypothesis testing of descriptive trends was not performed.

## 3 Results

**Figure 1:**
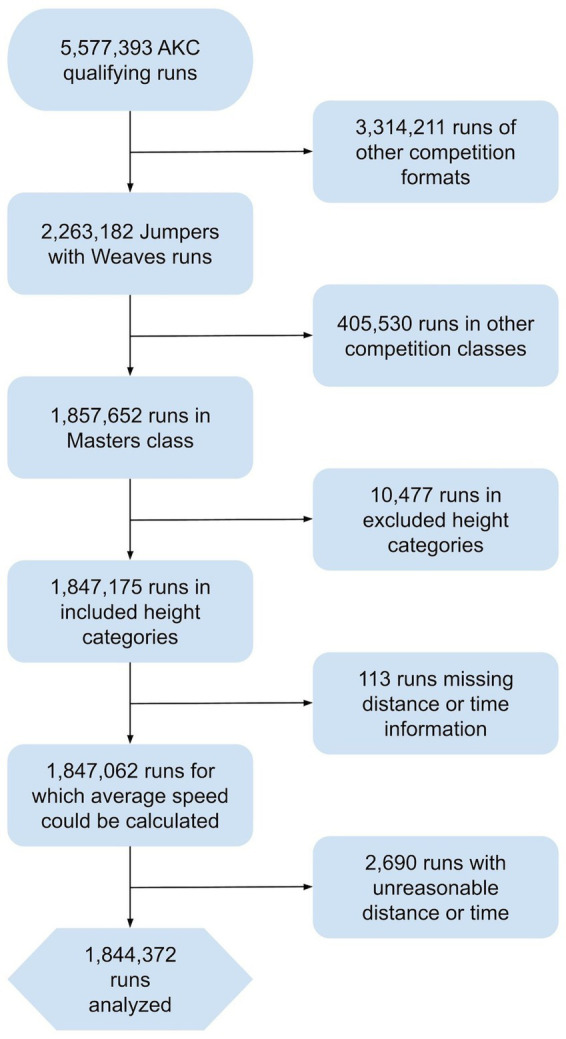
Flow diagram of inclusion/exclusion criteria.

The total number of qualifying runs analyzed after the inclusion and exclusion criteria were applied was 1,844,372 (Figure 1). Most of these runs were run in regular height categories (75%) with the remainder in preferred. A plurality of regular qualifying runs were from the 20 inch jump height category (31%), followed by 16 inch, 12, inch, 8 inch, and 24 inch in the regular classes. Preferred had similar demographics, with the most qualifying runs coming from the 16 inch jump height category (31%). The number of qualifying runs per year averaged 147,340, excluding the COVID-19 impacted year of 2020 which had only 76,298 qualifying runs.

The 1,844,372 total runs came from 35,540 unique dogs. The number of dogs in each starting cohort (the year of their first Masters qualifying run) averaged 2,121, again excluding 2020, which had a starting cohort of only 1,075 dogs. The number of qualifying runs per dog varied considerably, with a median of 24 (IQR: 7 to 66) and a mean of 51.9.

### 3.1 Aim 1: are qualifying runs increasing in speed?

**Figure 2:**
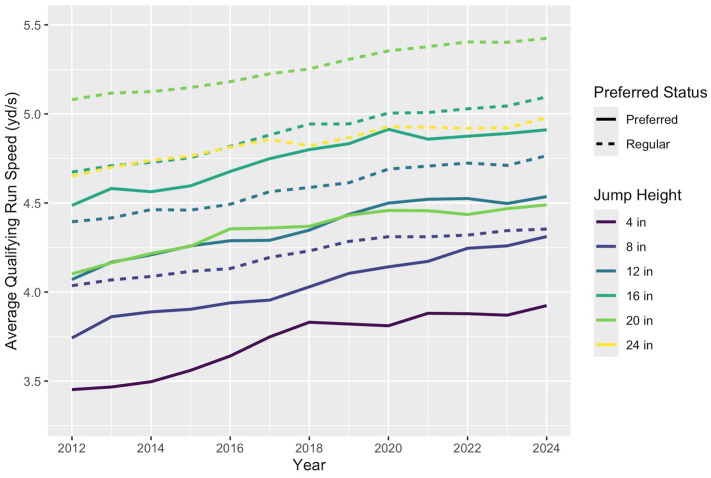
Line graph demonstrating linear increases in average qualifying run speed in all jump height and preferred status categories from 2012 to 2024. Regular runs had faster average qualifying run speeds than preferred runs across all shared jump height categories. This difference was particularly pronounced at the 20 inch jump height.

The empirical average qualifying run speed for each competition category increased approximately linearly from 2012 to 2024 (Figure 2). GEE estimated that average qualifying run speed increased linearly at an estimated rate of 0.0331 (95% CI: 0.0306, 0.0355) yards per second (yd/s) per year, adjusting for height category and preferred status. This estimated rate of change corresponds to an increase in average speed for regular status dogs competing at 20 inches (the most prevalent category) from 4.95 yd./s at the beginning of 2012 to 5.38 yd./s by the end of 2024. On an average course of 160 yards, this would correspond to an estimated 2.58 (95% CI: 2.40, 2.75) second decrease in qualifying run time over this period.

**Figure 3:**
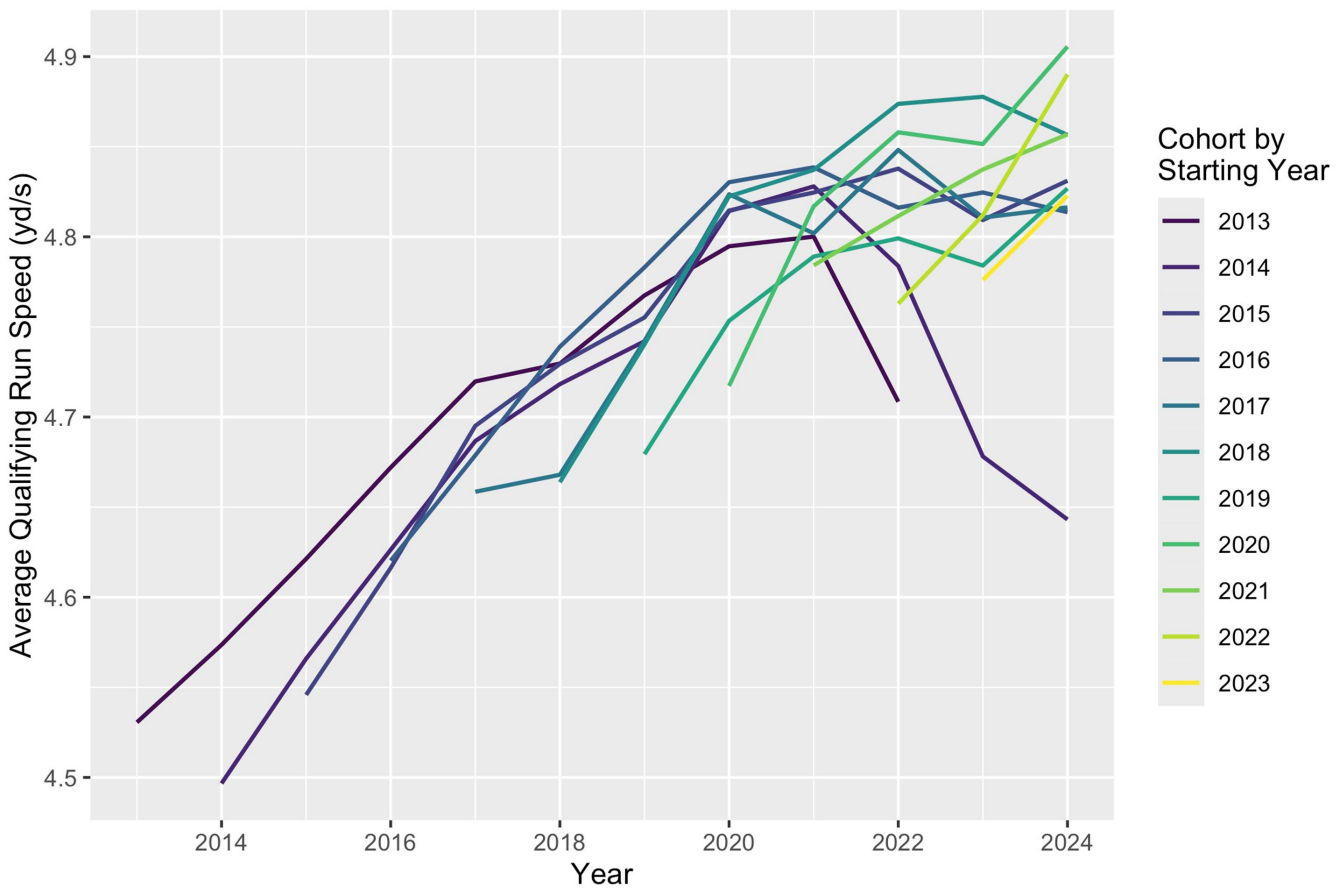
Average qualifying run speed by year and starting year cohort. Cohorts with later starting years have higher average speeds than earlier cohorts at the same point in their agility careers. Only cohort-years with at least 100 participating dogs were included.

Analysis of individual dog trajectories suggested that in a typical agility career, average speed increases for several years and then decreases until the dog is withdrawn from competition, though career trajectories vary greatly among individual dogs. This same general trend occurs when considering average qualifying speed dogs from the same starting year cohort, with average speed increasing for several years and then decreasing (Figure 3).

Notably, the initial starting speeds and the peak average speed were higher for dogs from more recent cohort years (Figure 3), which could partially explain the increase in average speed overall. For example, the average first-year speed among qualifying runs by dogs in the 2014 cohort was 4.50 yd./s, with a peak annual average speed of 4.83 yd./s observed 7 years into their career, in 2021. Among qualifying runs by dogs in the 2018 cohort, the average first-year speed was 4.66 yd./s and the peak annual average speed was 4.88 yd./s, observed 5 years into their career in 2023.

### 3.2 Aim 2: how do temporal trends in qualifying speed vary at different percentiles of competition?

**Figure 4:**
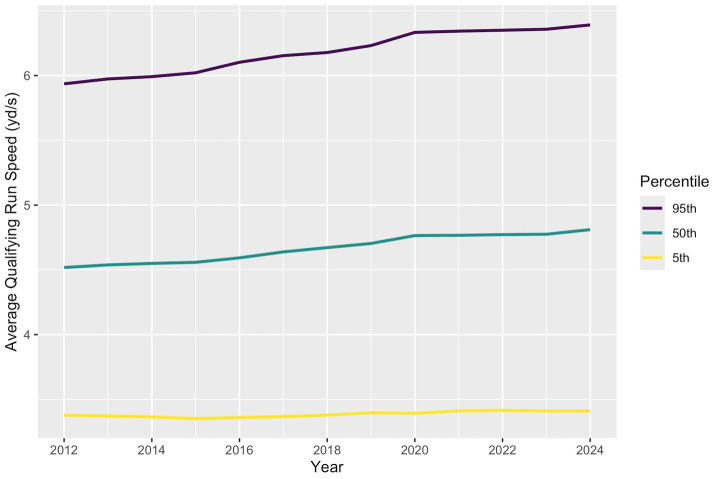
Median, 95% percentile, and 5% percentile of qualifying run speed from 2012 to 2024. Qualifying run speed increased approximately linearly over time at all percentiles considered, with greater increases in speed occurring at higher percentiles.

Qualifying run speed increased at all examined percentiles of competition (Figure 4). In the 1% of runs around the 95th percentile each year, average qualifying run speed increased linearly at an estimated rate of 0.0412 (95% CI: 0.0410–0.0414) yd./s per year. In the 1% of runs around the 50th percentile, speeds increased linearly by an estimated 0.0263 (95% CI: 0.0262–0.0264) yd./s each year. And in the 1% of runs around the 5th percentile, speeds increased by 0.00461 (95% CI: 0.00451–0.00471) yd./s per year. Notably, the speeds of the fastest qualifying runs increased most quickly and speeds of the slowest qualifying runs increased by a much smaller amount.

### 3.3 Aim 3: are competition characteristics or demographics shifting?

**Figure 5:**
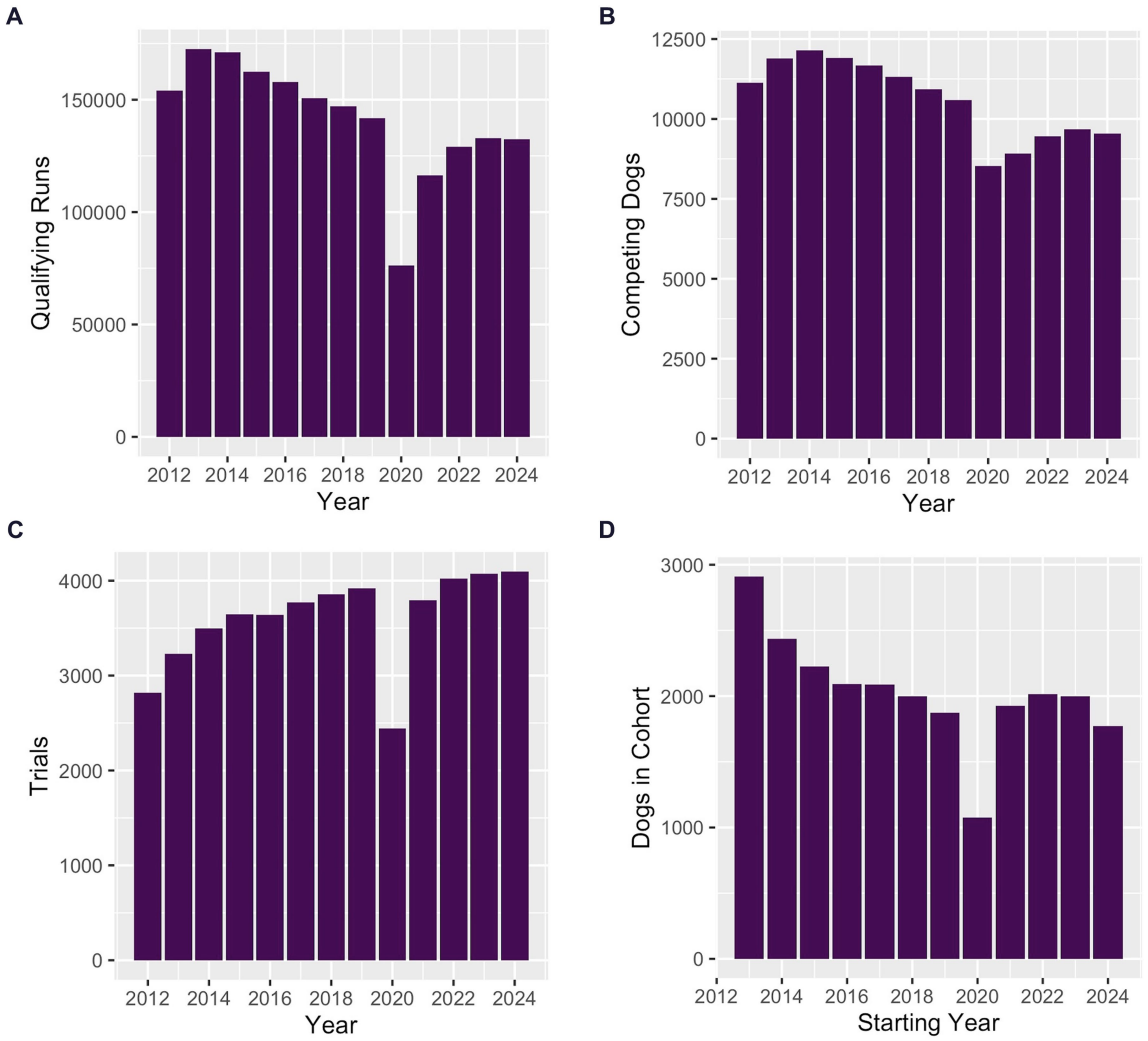
Bar graphs of the number of qualifying runs, competitors, trials, and new competitors. **(A–C)** From 2012 to 2024, the annual number of qualifying runs and the number of competing dogs both decreased, while the annual number of trials increased. **(D)** From 2013 to 2024, the number of dogs each year completing their first recorded qualifying run in JWW Masters declined.

From 2012 to 2024, the annual number of qualifying runs generally declined, as did the number of unique dogs competing each year (Figures 5A,B). Simultaneously, the number of unique trial days increased the number of opportunities for dogs to compete (Figure 5C). From 2013 to 2024, the number of unique dogs recorded for the first time as competing in JWW Masters decreased annually (Figure 5D). A major outlier in these trends was the year 2020, when many agility trials were canceled or attendance limited due to impacts of the COVID-19 pandemic.

**Figure 6:**
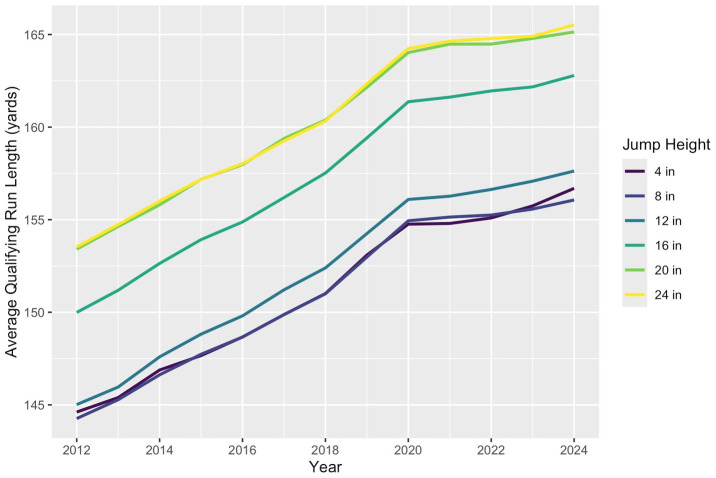
Average qualifying run course length over time, by jump height category. At all jump heights, course lengths increased from 2012 to 2024.

Average course length among qualifying runs increased from 2012 to 2024, mostly between 2012 and 2020. The rate of increase changed sharply in 2020, resulting in a much slower increase in average course length between 2020 and 2024 (Figure 6). This trend appeared across all jump height categories.

**Figure 7:**
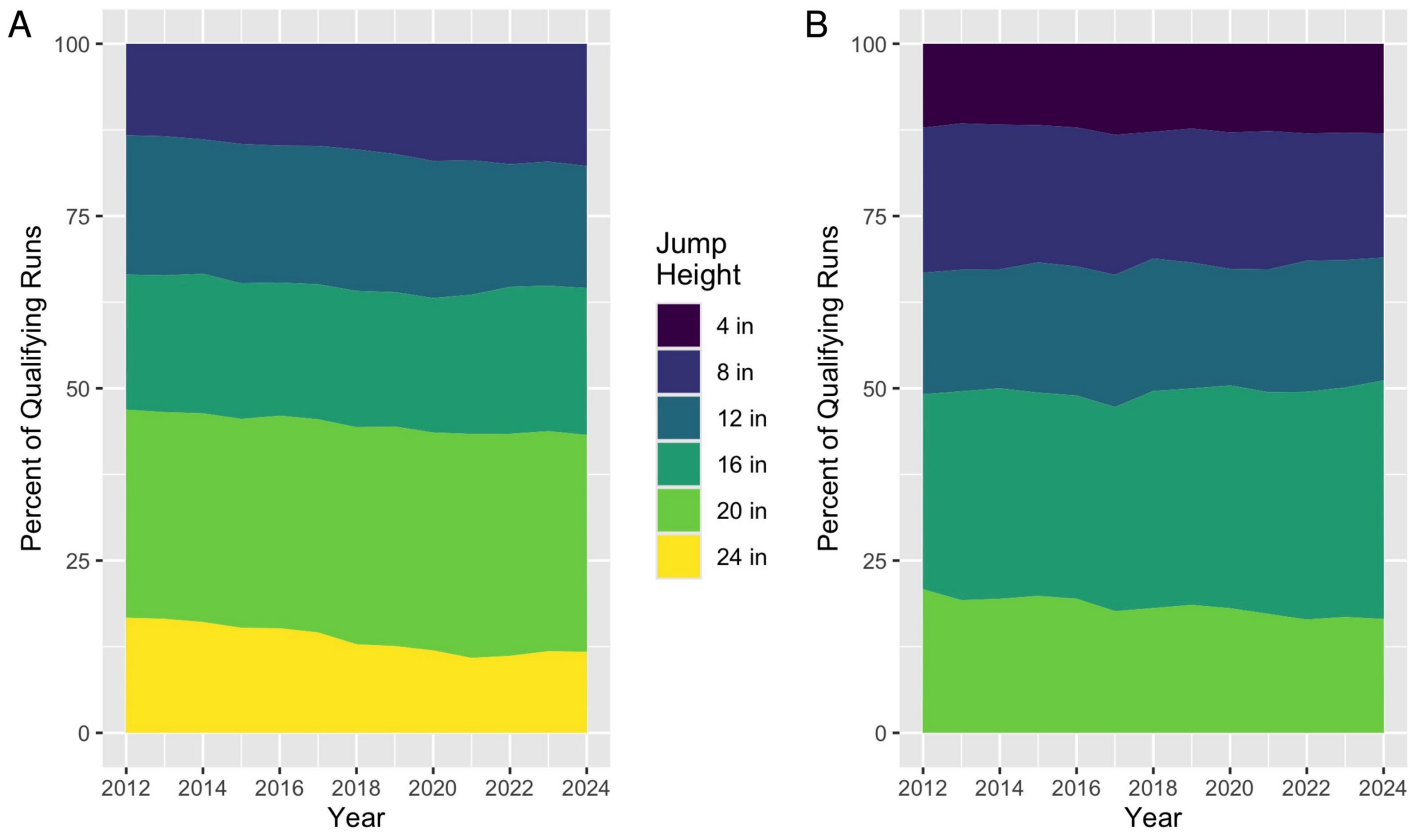
Jump height category over time, by preferred status. **(A)** There was a trend towards shorter jump height categories from 2012 to 2024 among qualifying runs with regular status (left). **(B)** Jump height categories remained more stable among runs with preferred status (right) over the same time period, with a slight increase in qualifying runs with the 16 inch jump height.

**Figure 8:**
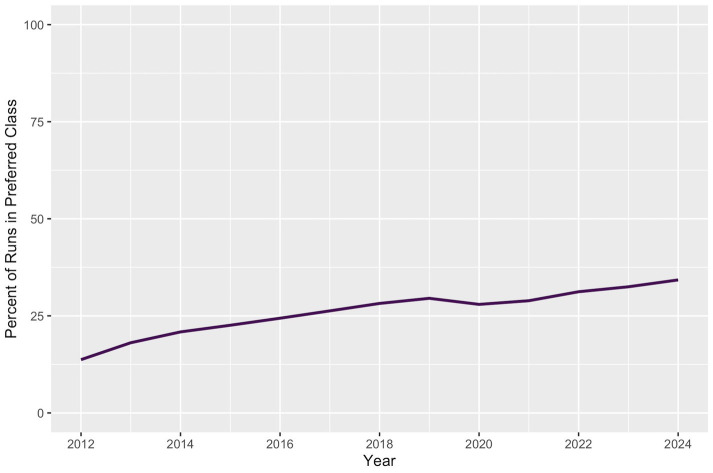
Percent of JWW Masters qualifying runs competed with preferred status. The proportion of qualifying runs with preferred status (as opposed to regular) increased approximately linearly from 2012 to 2024.

Demographically, there was a small increase in representation of shorter dogs among regular qualifying runs (Figure 7) and a substantial increase in the proportion of qualifying runs raced by dogs under the preferred designation (Figure 8) from 2012 (13.7%) to 2024 (34.3%).

**Figure 9:**
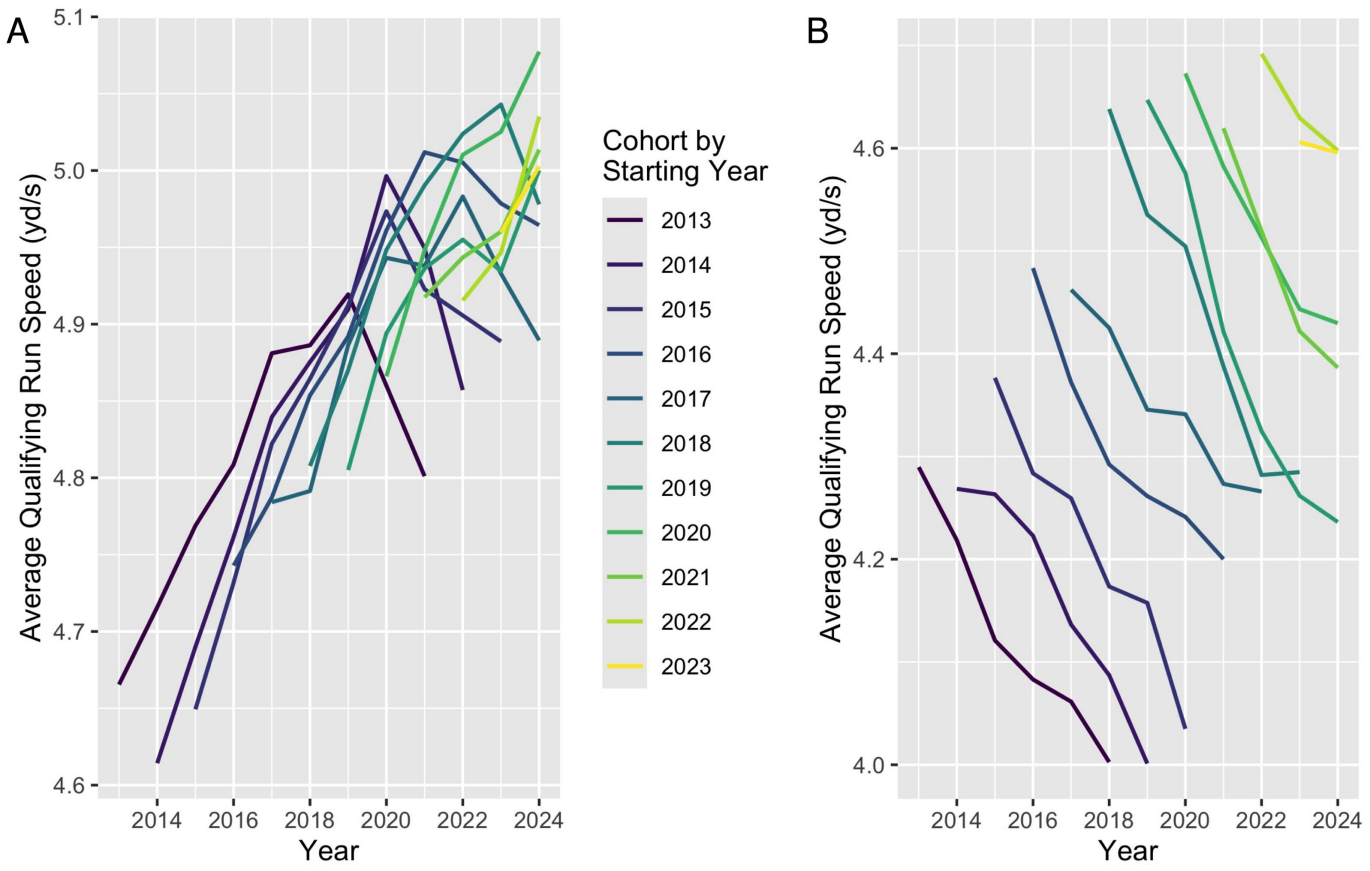
Average speed by cohort year among regular **(A)** and preferred **(B)** qualifying runs. In both the regular and preferred categories, average qualifying run speeds increased from cohort to cohort. **(A)** Average qualifying run speed in the regular category increased for several years, then decreased, while **(B)** average qualifying run speed in the preferred category decreased in every subsequent year of participation.

Among qualifying runs with regular status (the majority of runs), cohort average speed trajectories mirrored those in the full data set (Figure 9A). For qualifying runs raced with preferred status, average run speed also increased over time and average run speed in a cohort’s first year also increased for subsequent cohorts (Figure 9B). However, average qualifying run speed decreased yearly within preferred status cohorts, a different trend than that for regular status cohorts.

## 4 Discussion

Average qualifying run speed has increased over the past 13 years, consistent with anecdotal reports of increasing speed over time. This increase in speed appeared among regular and preferred runs at all jump heights, among fast, slow, and typical runs. While these findings do not establish a causal relationship, they motivate the need to explore whether rising speeds are associated with observed injury trends in agility dogs.

Why might we see this increase in average qualifying run speed over time? Although the distribution of jump height usage changed slightly over the period considered, the increase in speed cannot be easily explained by changes in jump height usage, since average qualifying run speed increased in every height category. However, other known temporal trends from 2012 to 2024 could have influenced speed. Surface type is correlated with speed in canine agility trials, with faster runs typically occurring on artificial turf and slower runs on sand or sand composite surfaces (9, 10). Over the past 15 years, there has been an anecdotal shift in trial surfaces in the United States, with increased utilization of artificial turf surfaces, and decreased utilization of sand and sand composite surfaces primarily found in equine arenas. This change could have directly contributed to faster course times by reducing the muscular effort and energy expenditure associated with running on more compliant surfaces such as sand (11, 12). The evolution of course design may also play a role in increasing speeds. Course design rule changes across organizations have prompted trends towards longer course lengths and increased obstacle spacing, which may allow for more extension and less frequent collection, thereby resulting in higher average speeds. ^7^^,^ ^8^ Changes in agility training and handling practices may also contribute to higher speeds. In particular, increased emphasis on precise cueing and efficient line management could allow teams to gain and maintain speed more effectively, although these trends have not been formally quantified. Purpose-breeding for agility may also contribute to increased speed over time, as selective breeding may emphasize traits such as speed, high-drive temperaments, and handler responsiveness. Over time, these selection pressures could shift the upper limits of performance within the agility dog population.

Analysis of individual dogs indicated that, in a typical agility career, average speed increases for several years before reaching a peak and then gradually declines until withdrawal from competition. The initial increase in speed likely reflects improvements in handler-dog communication, skill refinement, and increased fluency in navigating complex course demands as the team gains experience. This developmental phase parallels patterns observed in other athletic species, where early-career performance gains are associated with neuromuscular adaptation, technique optimization, and cumulative training effects (13–15). The subsequent decline in speed likely represents physiological and biomechanical aging processes. In humans and equine athletes, age-related physiological changes, including decreases in aerobic capacity (VO₂max), muscle mass, and strength, contribute to reduced performance, and similar trends are suspected in canine athletes (16–19). Also, with approximately 40% of agility dogs having sustained an injury during their careers, injury and development of chronic musculoskeletal conditions likely contribute to decline in speed and eventually retirement among a substantial portion of dogs (4). A combination of reduced physiological capacity, cumulative physical wear, and injury susceptibility likely underlies the late-career decline observed across cohorts. Although longitudinal physiological data has not been studied in dogs, the observed average speed trajectory is consistent with a model of athletic maturation followed by gradual decline with advancing age or accumulated workload, as seen in human athletes (13, 15, 18).

When dogs are grouped by the calendar year of their first qualifying run in Masters JWW, more recent cohorts demonstrate higher average speeds throughout their careers. This pattern suggests that dogs are entering Masters-level competition at faster speeds than those entering in earlier years. The reasons for this shift are likely multifactorial. Because individual dog ages were not available, it remains unknown whether dogs are entering the Masters level at similar ages across cohorts, limiting interpretation of whether this pattern is due to earlier sport specialization, older age at entry, or some other change in performance, health, or demographic trends. If dogs in recent cohorts are older and more experienced at entry, this could contribute to higher initial speeds independent of training intensity. If instead dogs are starting at comparable ages, the observed increase would more directly represent a true elevation in performance ability and speed. Incorporating age and longitudinal health data in future analyses will be essential for clarifying how these trends relate to athletic development, longevity, and potential injury risk. It may also shed some light on why we see a decrease in the number of dogs joining each new cohort.

It remains unclear whether newer cohorts are peaking earlier in their careers, as the most recent cohorts have not yet reached the full competitive lifespan observed in earlier cohorts. However, the slightly shorter time to peak in more recent cohorts (e.g., 5 years for the 2018 cohort compared to 7 years for the 2014 cohort) raises the possibility that dogs are achieving higher performance earlier in their athletic careers. While this could reflect improved training efficiency, it may also suggest increased physical strain or reduced longevity. This parallels observations in human and equine athletes where higher early-career workloads and sport specialization can be associated with shorter competitive longevity (20, 21). Whether these patterns correspond with earlier onset of musculoskeletal injury, degenerative change, or voluntary retirement from the sport warrants further investigation. Future studies incorporating longitudinal health and retirement data could help determine whether increased early-career performance intensity is associated with a shorter competitive lifespan or greater injury risk.

Average qualifying run speed increased approximately linearly over time at all percentiles considered, with greater increases occurring at the higher percentiles. That is, while qualifying runs across the performance spectrum have been increasing in speed on average, the rate of improvement has been greatest among the fastest qualifying runs. This widening performance gap suggests that elite-level teams are advancing at a pace that exceeds that of average or slower-performing teams. Increased competitiveness at the highest levels of the sport likely plays a substantial role. This selection pressure may drive disproportionate gains among top-performing dogs, particularly those competing at the highest levels. Other factors may include breeding and genetics, evolution of training and conditioning methods, and course design and surface changes that may allow performance optimization at the highest levels.

The steady increase in average course length from 2012 to 2020 is consistent with anecdotal observations of course design trends to increase spacing between obstacles. Increased spacing between obstacles may have been increasing average course length steadily until a new update to the AKC Agility Judge’s Guidelines^9^ was published in 2020. The new update included changes to increase obstacle spacing requirements, likely reflecting the trend toward increased obstacle spacing, despite the older guidelines. With the new guidelines, average course length has remained relatively stable since 2020, suggesting that the spacing between obstacles has been more stable since then. Throughout the time period considered, course length measurement was conducted by the judges, which may have contributed additional variability to the reported course lengths due to measurement error.

The proportion of qualifying runs competed with “preferred” status increased from 2012 to 2024. Because handlers can elect preferred status at any competition, this shift may reflect changes in strategy or concern for longevity. Dogs typically transition to preferred status as they age or develop physical limitations, as many believe that the reduced jump height lowers biomechanical load and may extend their competitive careers, though this assumption has not been validated (22). Some handlers may choose to switch dogs to preferred status earlier to proactively reduce injury risk, although the effect of reduced jump height on injury risk has not been directly evaluated. When dogs were assigned to preferred cohorts based on their first year of participation with preferred status, average qualifying preferred run speed decreased monotonically over time in all preferred cohorts, suggesting that most dogs exhibit a decline in performance once they have switched to competing with preferred status. Interestingly, average speed in the first year of a dog’s participation with preferred status increased in subsequent preferred cohorts, which could indicate that fitter, faster dogs are entering the preferred division sooner. However, interpreting these changes is complex: while lower jump heights can facilitate faster movement, slower runs are also more likely to qualify due to extended course times, potentially offsetting the effect on qualifying run averages.

The number of qualifying runs decreased over time, despite the concurrent increase in the number of trials offered. Such trends have been noted at national and international events across multiple organizations (4). This may reflect evolving course design, with judges introducing greater technical complexity and more handling challenges (e.g., more obstacle discriminations) to meet the demands of increasingly competitive teams. There is also a reduced margin of error at higher speeds, so the increasing speed over time could directly contribute to decreasing qualifying rates, particularly when combined with more challenging courses. However, fully evaluating trends in qualifying rates over time is challenging without individual level data on all attempted runs (qualifying or not qualifying).

The number of unique competing dogs also decreased, with fewer new dogs entering the JWW Masters division each year, despite an increasing number of trials. While it is possible that the population of competitors is shifting toward faster dogs, this trend alone is unlikely to explain the observed increase in average qualifying run speed. The slowest percentiles of qualifying runs demonstrated a much smaller increase in speed than the fastest percentiles of qualifying runs, suggesting that the change is not solely due to selection bias. Contributing factors to the decline in number of competitors may include earlier retirement of dogs, shifts in handler participation, and redistribution of competitors among a growing number of agility organizations and classes. The expansion of alternative venues such as UK Agility International, United States Dog Agility Association, and North American Dog Agility Council over the past decade may have drawn some competitors who previously participated in AKC Masters-level events.

A strength of this study is that data on all qualifying runs completed at AKC events were available for analysis, and that all AKC data are reported by trial officials, rather than owner-based reports. Unfortunately, only qualifying run data are available, so it is unknown how the trends of non-qualifying runs may be changing. Only data from JWW were analyzed, in order to remove the effect of contact obstacle performance variation (e.g., stopped versus running contacts) on the total course time and therefore on speed calculations. As a result, it is unknown how speed in Standard courses has changed over time. This is an important area for future research, as speed may influence contact obstacle performance safety and therefore injury risk. Masters level courses were chosen as the best representative of the dog’s total career, as they spend the longest portion of their career at the Masters level. However, this leaves a gap in our understanding of speed trends at lower levels of competition, and therefore likely a gap in our understanding of speed trends among young and inexperienced dogs. Since early agility development may influence long term health and performance among agility dogs, exploration of speed trends at competition levels below Masters could provide additional context for both changes in speed trends and injury frequency at the Masters level.

The primary contribution of this study is in identifying and quantifying trends in agility course speed over time, providing context for future investigations into potential associations between speed and injury. Progress in this area will depend on the systematic collection of detailed injury data from agility competitions, including both qualifying and non-qualifying runs. Developing a standardized injury reporting framework that can be integrated into existing competition data systems would represent an important step toward improving understanding and prevention of injuries in agility dogs. Additional research that would be useful for investigating the causes of the increase in qualifying run speeds includes analysis of changes in competition surfaces, course design and training methods, as well as analysis of age effects that may explain cohort trends over this time period.

## Data Availability

The data analyzed for this study can be found on the AKC Event Search and Results webpage (webapps.akc.org/event-search/).

## Footnotes

1
akc.org/sports/agility

2
akc.org/sports/agility/pdf/rulebooks/REAGIL.pdf

3
https://images.akc.org/pdf/events/agility/0217_Equipment_Changes_Chapter_3_insert_Corrected_.pdf

4
https://www.akc.org/expert-advice/news/akc-agility-suspends-use-of-the-collapsed-tunnel/

5 webapps.akc.org/event-search/

6 On January 3, 2024, AKC adjusted the greatest allowable standard course times for the JWW Masters division when they published their 2024 course regulations. For trials prior to this change, the previous standard course time calculation was used to determine exclusion. For trials following this change, the updated standard course time calculation method was used.

7
https://www.royalkennelclub.com/about-us/resources/media-centre/2016/october/minimum-distance-between-agility-obstacles-increased

8
https://images.akc.org/pdf/events/agility/judges_guidelines_2010.pdf

9
https://images.akc.org/pdf/rulebooks/REAJG1.pdf
